# Acupuncture for polycystic ovarian syndrome: A systematic review and meta-analysis

**DOI:** 10.1097/MD.0000000000007066

**Published:** 2017-06-08

**Authors:** Junyoung Jo, Yoon Jae Lee, Hyangsook Lee

**Affiliations:** a Department of Korean Gynecology, Conmaul Hospital of Korean Medicine; b Department of Korean Gynecology, Jaseng Hospital of Korean Medicine; c Acupuncture and Meridian Science Research Center, College of Korean Medicine, Kyung Hee University, Seoul, Korea; d Australian Research Center in Complementary and Integrative Medicine, Faculty of Health, University of Technology Sydney, Sydney, Australia.

**Keywords:** acupuncture, menstrual cycle, meta-analysis, ovulation, polycystic ovarian syndrome, randomized controlled trial

## Abstract

**Background:**

This systematic review aimed at summarizing and evaluating the evidence from randomized controlled trials (RCTs) using acupuncture to treat polycystic ovarian syndrome (PCOS), specifically focusing on ovulation rate, menstrual rate, and related hormones.

**Methods:**

Fifteen databases were searched electronically through February 2016. Our review included RCTs of women with PCOS; these RCTs compared acupuncture with sham acupuncture, medication, or no treatment. Two reviewers independently extracted data. Data were pooled and expressed as mean differences (MDs) for continuous outcomes and risk ratios for dichotomous outcomes, with 95% confidence intervals (CIs) using a random-effects model.

**Results:**

We found a low level of evidence that acupuncture is more likely to improve ovulation rate (MD 0.35, 95% CI: 0.14–0.56) and menstruation rate (MD 0.50, 95% CI: 0.32–0.68) compared with no acupuncture. We found statistically significant pooled benefits of acupuncture treatment as an adjunct to medication in luteinizing hormone (LH), LH/follicular stimulating hormone (FSH) ratio, testosterone, fasting insulin, and pregnancy rates, but the level of evidence was low/very low.

**Conclusion:**

There is limited evidence to judge the efficacy and safety of acupuncture on key reproductive outcomes in women with PCOS. Large-scale, long-term RCTs with rigorous methodological input are needed.

## Abstract 2

Supplemental Digital Content is available in the text

## 1 Introduction

Polycystic ovarian syndrome (PCOS) is diagnosed on the basis of oligo-ovulation or anovulation, hyperandrogenism, and the presence of polycystic ovaries.^[1]^ The prevalence of PCOS is as high as 15% when the Rotterdam criteria are applied.^[2]^ PCOS is estimated to account for 70% to 90% of ovulatory disorders.^[3]^

No single etiologic factor fully accounts for the spectrum of abnormalities in PCOS.^[4]^ The most obvious neuroendocrine feature in PCOS is increased luteinizing hormone (LH) pulsatility, with relatively low follicular stimulating hormone (FSH) secretion.^[5]^ One of the common features of PCOS is insulin resistance, reported in 62% to 95% of women with PCOS.^[6]^ Elevated androgen level, another common feature of PCOS, affects around 60% to 80% of women with PCOS and can produce clinical signs like hirsutism, acne, and alopecia.^[7]^

Clomiphene citrate (CC), a selective estrogen-receptor modulator, has been used as 1st-line treatment for PCOS for decades.^[8]^ CC is not without drawbacks, however, including its overall poor efficacy, a relatively high multiple-pregnancy rate (3%–8%), and side effects such as mood changes and hot flushes.^[8]^

Acupuncture involves the insertion of needles into specific anatomical points (termed acupoints) and has been used in eastern Asian countries for thousands of years. Recently, the use of acupuncture in reproductive endocrinology and infertility has gained increased popularity worldwide.^[9]^ Several clinical and animal experimental studies indicate that acupuncture is beneficial for ovulatory dysfunction in PCOS.^[5]^ Acupuncture has also been reported to potentially improve insulin sensitivity and to decrease testosterone in patients with PCOS.^[10,11]^

Recently, several systematic reviews on acupuncture for PCOS were published.^[12–14]^ However, there are some discrepancies among these studies and outcomes. The 1st meta-analysis by Qu et al (2016) focused on the recovery of menstrual cycles and hormone levels, but it was based on only 9 randomized controlled trials (RCTs) with a total of 531 participants. The 2nd review by Wu et al (2016) looked at 31 RCTs with 2371 subjects, but it did not attempt a meta-analysis. The 3rd Cochrane review by Lim et al (2016) included only 5 RCTs with 413 participants and focused on live birth and ovulation only. Thus, no one review comprehensively included all the available studies nor performed meta-analyses of important outcomes including menstruation cycles, pregnancy, and hormonal changes.

Therefore, this systematic review aimed at summarizing and evaluating the currently available evidence from RCTs of acupuncture to treat PCOS, specifically focusing on ovulation rate, menstrual rate, and related hormones.

## 2 Materials and methods

The protocol for this systematic review was registered (CRD42015016485) and the review was conducted and reported as outlined in the Preferred Reporting Items for Systematic Reviews and Meta-Analyses statement.^[15]^

### 2.1 Search strategies

We searched databases for relevant studies published through February 2016, comprising 4 international, 3 Chinese, 6 Korean, and 2 Japanese databases. The detailed search strategies are provided in Appendix S1. References of relevant publications (eg, gynecology textbooks, complementary and alternative medicine textbooks, clinical guidelines, or reviews of infertility) were also hand-searched. No language restrictions were imposed.

### 2.2 Study selection

Our review included RCTs of women with PCOS; these RCTs compared acupuncture with sham acupuncture, medication, or no treatment. The detailed study selections are provided in Appendix S2.

### 2.3 Data extraction

All studies were reviewed and selected independently by 2 reviewers (JJ and YJL). The titles and abstracts were reviewed and articles that did not fit the eligibility criteria were excluded. If the title or abstract appeared to meet the eligibility criteria, the full-texts of the articles were obtained for further evaluation. Discrepancies between the reviewers were resolved by consensus among all 3 reviewers. The independent reviewers extracted and tabulated data using a standardized data extraction form, with disagreements resolved by discussion with the corresponding author (HL). The form included information pertaining to first author, study design, quality of methods, language of publication, country where the trial was conducted, inclusion/exclusion criteria, PCOS diagnostic criteria used, number of participants allocated to each group, acupuncture intervention details, comparison groups, outcome measures, follow-up periods, and reported adverse events associated with acupuncture. When studies reported outcomes at more than 1 time point, a similar measurement point in other studies was taken for analysis. If the data in an article were insufficient or ambiguous, 1 author (YJL) contacted the corresponding author by e-mail to obtain further information.

### 2.4 Risk of bias assessment

We evaluated the risk of bias among the included studies using the risk of bias assessment tool by the Cochrane Collaboration.^[16]^ The criteria consist of 7 items related to selection bias (random sequence generation and allocation concealment), performance bias (blinding of participants and personnel), detection bias (blinding of outcome assessment), attrition bias (incomplete outcome data), reporting bias (selective outcome reporting), and other source of bias. Each study was assigned “yes” for a low risk of bias, “no” for a high risk of bias, or “unclear” for an unclear risk of bias for each item. Any discrepancies between the 2 authors were resolved by discussion with the corresponding author (HL) until consensus was reached.

### 2.5 Data synthesis

Statistical analyses were performed with the Review Manager program (Version 5.3 Copenhagen: The Nordic Cochrane Centre, The Cochrane Collaboration, 2014) and Stata (StataCorp 2015; Release 14. College Station, TX: StataCorp LP). Trials were combined according to the type of intervention, outcome measure, and/or control. Data were pooled and expressed as mean difference (MD) for continuous outcomes and risk ratio for dichotomous outcomes with 95% confidence intervals (CIs) using a random-effects model to incorporate expected heterogeneity. Heterogeneity among studies was assessed using χ^2^ test with a significance level of *P* < .1 and *I*^2^ statistic.^[17]^ The *I*^2^ statistic indicates the proportion of variability among trials that is not explained by chance alone and we considered an *I*^2^ value >50% to indicate a substantial heterogeneity.^[17,18]^ If a substantial heterogeneity was detected, we explored sources of heterogeneity by subgroup analysis. Subgroup analyses were attempted according to type of control (eg, medication type). If some factor (eg, large methodological and/or clinical difference among trials) was found, we did not conduct subgroup analysis or data synthesis, but reported a narrative description of the included studies. When there were more than 10 trials in the analysis, reporting biases such as publication bias were assessed by funnel plots. If asymmetry is suggested by a visual inspection, we performed exploratory analyses using Egger method.^[17]^

### 2.6 Dealing with missing data

As much as possible, data were analyzed using an intention-to-treat basis, and attempts were made to obtain missing data from the original investigators. When these attempts were not successful, we did not impute data for missing data, but only analyzed available data.

### 2.7 Level of evidence

Grading of Recommendations, Assessment, Development and Evaluation (GRADE) was used to assess the level of evidence and summarize each outcome.^[19]^ The level of evidence was categorized into 4 levels: high, moderate, low, or very low quality. The GRADE pro software (version 3.6.1 for Windows, Grade Working group) was used.

## 3 Results

**Figure 1:**
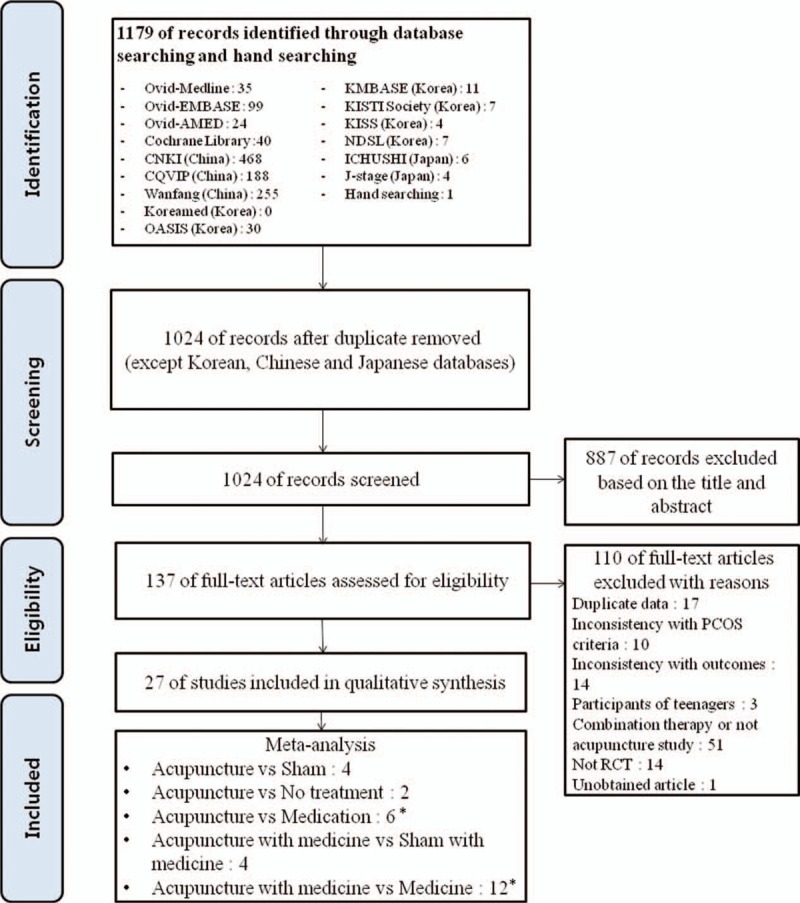
PRISMA flow diagram of literature searching and article selection process. ∗From 3-arm study. PCOS = polycystic ovarian syndrome, PRISMA = Preferred reporting items for systematic reviews and meta-analyses, RCT = randomized controlled trials, Sham = sham acupuncture.

Our initial search identified 1179 records, of which 1024 articles were screened. We excluded 887 articles based on the title and abstract, and retrieved 137 articles for more detailed evaluation. Of these, 27 RCTs were included (Fig. 1).^[15]^

### 3.1 Characteristics of the included studies

#### 3.1.1 Study design

Of 27 studies, 23 originated from China and were all published in Chinese^[20–41]^ except 1 trial that was published in English.^[42]^ Two studies were performed in Sweden,^[10,43]^ 1 in America,^[44]^ and 1 in Australia and China.^[45]^ Although 7 articles^[21,22,33,36–38,40]^ were master's theses, 20 studies were published in peer-reviewed journals.

#### 3.1.2 Participants

A total of 2093 participants were enrolled in the 27 studies with sample sizes ranging from 25 to 251. Calculations of sample size and statistical power were reported in only 3 studies.^[10,43,44]^ All participants were diagnosed with PCOS according to Rotterdam criteria.^[1]^ Eight trials involved women with PCOS and subfertility together.^[20–22,31,32,34,36,38]^ Although 7 studies were conducted only in patients with obesity-type PCOS (body mass index (BMI) ≥25 kg/m^2^),^[20,26,32,34,37,41,42]^ 7 did not report BMI.^[20,21,25,30,31,39,40]^ Baseline characteristics among groups were reported as comparable in each study.

#### 3.1.3 Interventions

Twelve trials tested the effectiveness of acupuncture alone^[10,20,24,29–31,37,42–45]^ and the others used acupuncture as an adjunct to CC,^[21,22,28,33,35,36,38]^ Chinese herbal medicine (CHM),^[26,27,40]^ metformin,^[20,32]^ Diane-35, or combinations of these.^[15,25,34,39,41]^ Nineteen trials used manual acupuncture^[20–22,25–32,34–36,39–42,45]^ and the others used manual acupuncture with electrical stimulation, that is, electroacupuncture (EA).^[10,20,24,33,37,38,43,44]^ Acupuncture interventions varied in acupoint selection, frequency of treatment, and number of treatment sessions across studies. The duration of therapy ranged from 10 weeks to 6 months. The characteristics of the included studies are presented in Table 1 and more detailed information on acupuncture interventions are provided in Table S1.

**Table 1:**
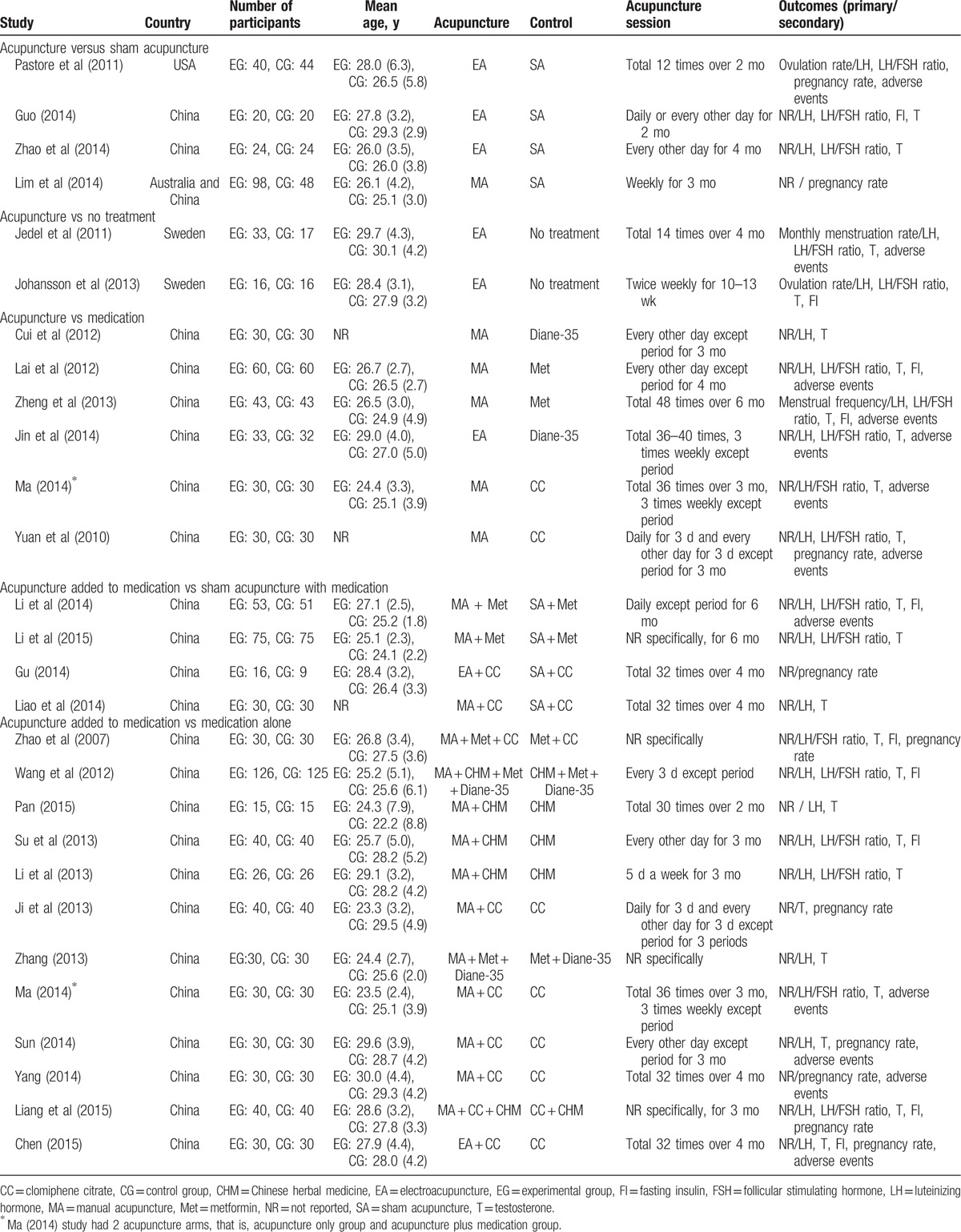
Characteristics of the included studies.

#### 3.1.4 Outcomes

For primary outcomes, 2 studies reported ovulation rate^[10,44]^ and another 2 studies reported menstruation rate.^[42,43]^ For secondary outcomes, LH,^[10,20,21,24–27,29–35,37,39,40,42–44]^ LH/FSH ratio,^[10,20–22,24–27,29,31,32,34,37,39,41–44]^ testosterone,^[10,20–22,24–35,37,39–43]^ fasting insulin levels,^[10,20,25,26,29,33,34,37,41,42]^ and pregnancy rate^[21,28,31,33,34,36,38,41,44,45]^ were reported. One study^[28]^ established pregnancy by blood and urine human chorionic gonadotropin testing. Another study^[36]^ determined pregnancy by ultrasound. In 2 studies,^[44,45]^ pregnancy was established by participant self-report. Six studies^[21,31,33,34,38,41]^ did not document clearly the diagnostic criterion (ie, biochemical or clinical) for pregnancy. Eleven studies reported adverse events.^[20–22,24,29,31,33,36,42–44]^ In 7 of the 11 studies, no adverse events were reported in patients receiving acupuncture.^[20,21,24,29,31,36,42]^ Sixteen studies did not report adverse events.^[10,20,25–28,30,32,34,35,37–41,45]^

### 3.2 Risk of bias in the included studies

**Figure 2:**
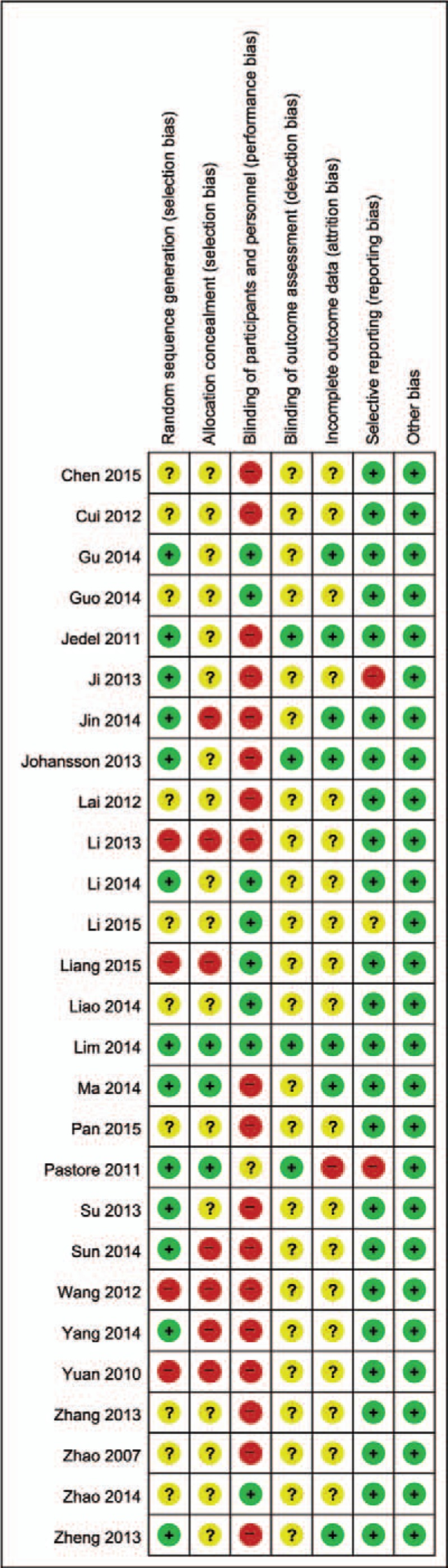
Risk of bias assessed using the Cochrane “Risk of bias” tool. +, High risk of bias; ?, unclear risk of bias; and −, low risk of bias.

A summary of the risks of bias is provided in Fig. 2 and the authors’ judgments on risk of bias are provided in Appendix S3.

### 3.3 Effects of acupuncture

We summarized the outcomes according to the following categories, based on the type of control group: acupuncture versus sham acupuncture; acupuncture versus no treatment; acupuncture versus medication; acupuncture with medication versus sham acupuncture with medication; and acupuncture with medication versus medication alone (Table 2).

**Table 2:**
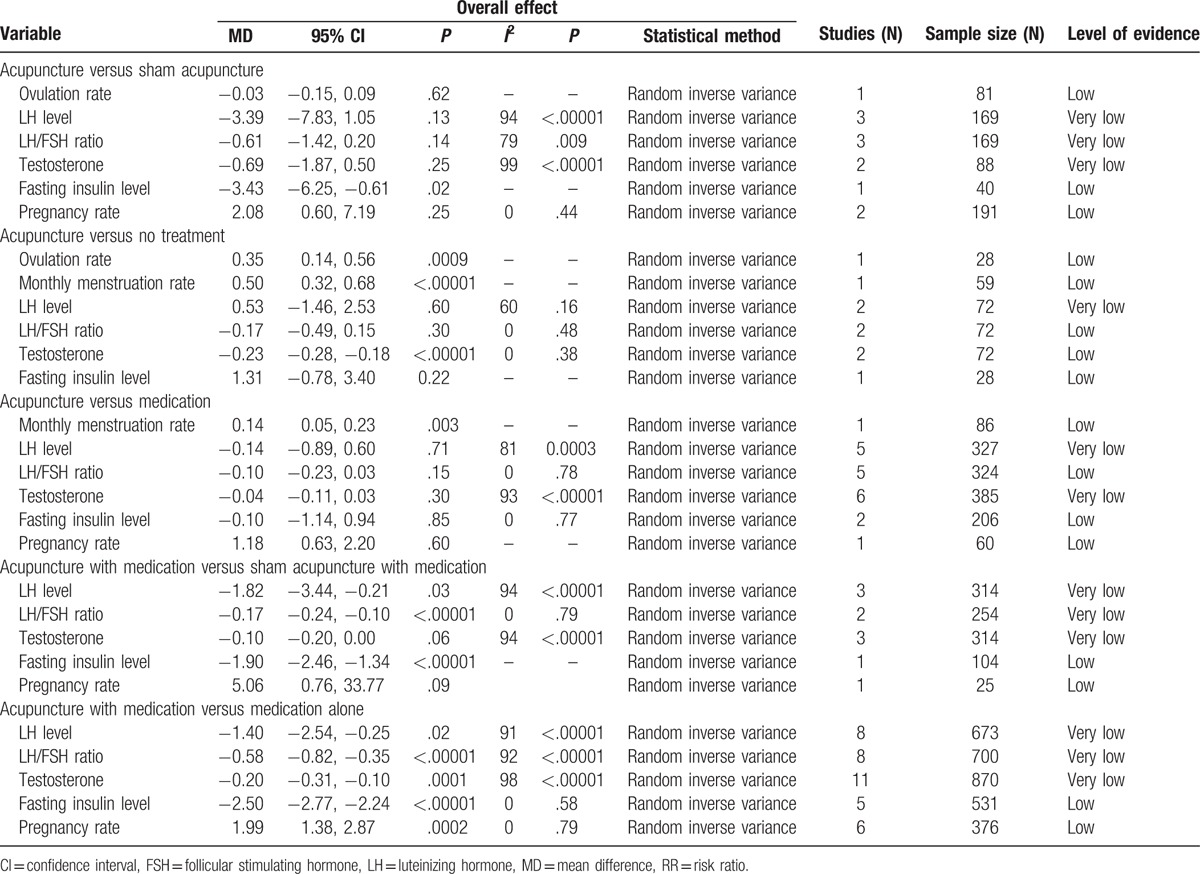
Meta-analysis of outcomes and level of evidence.

#### 3.3.1 Acupuncture versus sham acupuncture (4 studies)

Outcomes: There were no studies that reported menstruation rate. There was evidence of an improvement in fasting insulin levels between women who received true versus sham acupuncture (MD −3.43, 95% CI −6.25 to −0.61, *P* = .02).^[37]^ There were no significant differences in other outcomes (Table 2).

Adverse events: One study^[44]^ reported 1 participant having a back spasm during an acupuncture session, and a subsequent evaluation by a physician outside the study team determined that the spasm was unrelated to the treatment. The others^[20,37,45]^ did not report adverse events.

#### 3.3.2 Acupuncture versus no treatment (2 studies)

Outcomes: There was evidence of improvement in ovulation rates, monthly menstruation rates, and testosterone levels in the acupuncture group compared to no treatment (Table 2).^[10,43]^ There were no studies that reported pregnancy rate. No significant differences in other outcomes emerged (Table 2).

Adverse events: One study^[43]^ mentioned that 3 participants had adverse events (isolated redness and subsequent hematomas) after 1 of the 14 low-frequency EA treatments. One participant reported dizziness and 1 reported nausea after 1 low-frequency EA treatment. No long-term adverse events occurred in the low-frequency EA group. Another study^[10]^ did not report adverse events.

#### 3.3.3 Acupuncture versus medication (6 studies)

Outcomes: There were no studies that reported ovulation rate. There was evidence of an improvement in monthly menstruation rates in women who received acupuncture compared to those who received metformin (MD 0.14, 95% CI 0.05 to 0.23, *P* = .003).^[42]^ The pooled results from 6 studies^[22,24,29–31,42]^ showed no significant difference in testosterone levels between the acupuncture and medication groups (MD −0.04, 95% CI −0.11 to 0.03, *P* = .30, *I*^2^ = 93%) with considerable heterogeneity. We conducted a subgroup analysis according to the control type, and the pooled results from 2 studies^[29,42]^ comparing acupuncture with metformin showed a significant difference in testosterone levels in the acupuncture group compared to the medication group (MD −0.13, 95% CI −0.21 to −0.05, *P* = .002, *I*^2^ = 0%, Fig. S1). There were no significant differences in other outcomes (Table 2).

Adverse events: All but 1 study^[30]^ reported adverse events. One patient in the Diane-35 group^[24]^ had a gastrointestinal problem, while no adverse events occurred in the acupuncture group. Metformin groups from 2 studies^[29,42]^ reported gastrointestinal problems such as nausea, vomiting, and diarrhea, whereas no adverse events occurred in the acupuncture group. Four patients among 30 who received CC treatment^[31]^ reported mild nausea, but no adverse events occurred in the acupuncture group. In another study,^[22]^ 2 among 30 patients had mild bleeding at the site of needling in the acupuncture group, and 5 patients among 28 in the CC group had gastrointestinal problems.

#### 3.3.4 Acupuncture with medication versus sham acupuncture with medication (4 studies)

Outcomes: There were no studies that reported ovulation rates and monthly menstruation rates (per woman). There was evidence of an improvement in LH levels, LH/FSH ratios,^[20,32]^ and fasting insulin levels^[20]^ in the true acupuncture with medication group compared to the sham acupuncture with medication group (Table 2). The pooled results from 3 studies^[20,32,35]^ showed a significant difference in LH levels in the true acupuncture with medication group compared to the sham acupuncture with medication group (MD −1.82, 95% CI −3.44 to −0.21, *P* = .03, *I*^2^ = 94%, Table 2). When we conducted a subgroup analysis according to the control medication type, acupuncture plus metformin^[20,32]^ showed a significant difference in LH levels compared with sham acupuncture with metformin (MD −0.99, 95% CI −1.35 to −0.63, *P* < .00001, *I*^2^ = 0%, Fig. S2). The pooled results in testosterone levels^[20,32,35]^ showed a marginally significant difference between true acupuncture with medicine and sham acupuncture with medicine (MD −0.10, 95% CI −0.20 to 0.00, *P* = .06, *I*^2^ = 94%, Table 2). A subgroup analysis revealed that true acupuncture with metformin^[20,32]^ also showed a marginally significant difference in testosterone levels compared with sham acupuncture with metformin (MD −0.05, 95% CI −0.10 to 0.00, *P* = .04, *I*^2^ = 54%, Fig. S2). There was no evidence of a difference in pregnancy rates compared with sham acupuncture with CC (Fig. S2).

Adverse events: One study^[20]^ reported that 22 patients (43.14%) in the sham acupuncture with metformin group had nausea or vomiting, mild diarrhea, and slight dizziness or weakness, while 18 patients (33.96%) in the true acupuncture with metformin group experienced these events. Three studies^[32,35,37]^ did not report adverse events.

#### 3.3.5 Acupuncture with medication versus medication alone (12 studies)

Outcomes: There were no studies that reported ovulation rates and monthly menstruation rates (per woman). There was evidence of an improvement in LH levels, LH/FSH ratios, testosterone levels, fasting insulin levels,^[25,26,33,34,41]^ and pregnancy rates^[21,28,33,34,36,41]^ (Table 2). The pooled results^[21,25–27,33,34,39,40]^ showed a significant improvement in LH levels in the true acupuncture with medication group compared with the medication alone group (MD −1.40, 95% CI −2.54 to −0.25, *P* = .02, *I*^2^ = 91%). A subgroup analysis according to the control group revealed that acupuncture added to combined medication (CHM plus CC),^[34]^ (CHM plus metformin and Diane-35),^[25]^ (metformin with Diane-35)^[39]^ only showed a significant difference in LH levels when compared with combined medication alone (MD −1.88, 95% CI −2.55 to −1.21, *P* < .00001, *I*^2^ = 47%, Fig. S3). The pooled results showed acupuncture produced a significant improvement in LH/FSH ratios^[21,22,25–27,34,39,41]^ and testosterone levels^[21,22,25–28,33,34,39–41]^ with considerable heterogeneity (Fig. S3). We conducted a subgroup analysis according to the control group, but heterogeneity was not resolved.

Adverse events: One study^[33]^ reported that 1 patient among 30 in the acupuncture group had mild pain at the site of needling. In another study,^[22]^ 3 among 29 patients had mild bleeding at the site of needling in the acupuncture combined with CC group, and 5 patients among 28 in the CC group had gastrointestinal problems, whereas there were no gastrointestinal problems in the acupuncture group. Two studies^[21,36]^ reported that there were no serious adverse events. Eight studies^[25–28,34,39–41]^ did not report adverse events.

### 3.4 Levels of evidence

The levels of evidence as determined by GRADE were found to be from very low to low (Table 2). Most of the studies were classified as having either an unclear or a high risk of selection bias, performance bias, and attrition bias, so all outcomes were initially downgraded in risk of bias domain. In addition, all outcomes were downgraded in the imprecision domain, due to small sample size that was far from optimal information size. The inconsistency domain was downgraded for unexplained heterogeneity in the outcomes: LH levels, LH/FSH ratios, and testosterone levels in the acupuncture versus sham acupuncture group; LH levels in the acupuncture versus no treatment group; LH levels, testosterone levels in the acupuncture versus medication group, LH levels, LH/FSH ratios, testosterone levels in the acupuncture plus medication versus sham acupuncture plus medication group; and LH levels, LH/FSH ratios, testosterone levels in the acupuncture plus medication versus medication alone group (Table 2).

## 4 Discussion

### 4.1 Summary of main findings

The objective of this review was to summarize and evaluate acupuncture treatment to improve ovulation and menstruation rates and other hormonal changes, in women with PCOS. We found a low level of evidence that acupuncture is more likely to improve ovulation and menstruation rates compared to not receiving acupuncture. When compared with metformin, acupuncture improves menstruation rates but the level of evidence is also low. We found statistically significant benefits of acupuncture treatment for up to 4 months as an adjunct to medication, seen in LH levels, LH/FSH ratios, testosterone levels, fasting insulin, and pregnancy rates, but the level of evidence is very low or low, mainly due to high risk of bias and heterogeneity. To date the evidence on acupuncture for PCOS remains largely inconclusive as the studies we reviewed tested different acupuncture protocols against various control types and the reported outcomes varied greatly. Acupuncture seems to be associated with few adverse events. Reported adverse events, such as needling pain, were mild and transient; there were no serious adverse events leading to withdrawals from the study.

### 4.2 Applicability of the current evidence

The included studies poorly addressed ovulation and menstruation rates, the primary outcomes in our review. Only 4 among 27 studies reported ovulation and/or menstruation rates. We found that acupuncture significantly improved monthly menstrual rates in comparison with no treatment or medication only, while ovulation rates were significantly improved by acupuncture only when it was compared with no treatment, but not with sham acupuncture. There may be 2 different interpretations for this. Acupuncture works mainly via a placebo effect^[46]^; or the adopted sham acupuncture control may not be completely inert.^[5,44,47]^ It is premature to determine which interpretation is valid as there are too few studies to make an evidence-based decision.

The optimal acupuncture treatment is a complex issue involving a range of contributing factors,^[48]^ for example, number of sessions, acupoint specificity and selection, stimulation methods, and the practitioner's expertise. In the study comparing acupuncture with a sham control where no difference was detected in ovulation rates,^[44]^ women received 12 acupuncture sessions over 8 weeks and the needle placement and stimulation was identical in both true and sham acupuncture groups. In another study,^[10]^ where needles were placed similarly as in the previous study,^[44]^ women received acupuncture twice weekly during the study period, thus an increased dose of acupuncture treatment was tested. Women allocated to the acupuncture group had a higher ovulation frequency compared with the no treatment group, indicating a dose–response effect as well as an augmented acupuncture effect.^[5]^ This tendency was more evident in other study^[20]^ where acupuncture treatment was conducted every other day for 4 months. This suggests that different acupuncture doses exert different treatment effects, and therefore, exploring the optimal acupuncture treatment intervention for PCOS should be preconditions of any future trials.

Although limited by heterogeneity across studies, we found that acupuncture adjuvant to other active medications could affect hormonal levels such as LH, LH/FSH ratio, testosterone, and fasting insulin. Neuroendocrinological mechanisms of acupuncture have been extensively studied not only in pain research^[49,50]^ but also in reproductive medicine.^[51]^ Acupuncture is also known to modulate hypothalamic-pituitary-ovarian axis, which can affect menstruation cycles.^[52]^ Given that acupuncture stimulates pituitary beta-endorphin production, which has a tonic inhibitory effect on gonadotropin-releasing hormone pulse generator and pituitary LH secretion, it is possible that acupuncture may reduce ovulatory dysfunction and thus decrease the secretion of ovarian androgens in women with PCOS.^[51]^ Considering detected heterogeneity from our analysis, known variability in hormonal levels, the poor standardization of assays, and the specific PCOS phenotypic features,^[53,54]^ however, the currently available data from RCTs has yet to allow us to draw any firm conclusion whether acupuncture affects hormonal levels, thus recovering ovulatory function and menstruation cycle in women with PCOS.

The pooled data showed that acupuncture significantly increased pregnancy rates when added to medication compared to medication alone. However, as the definition of pregnancy (ie, clinical pregnancy determined by ultrasound) was not uniform across studies, this finding needs to be confirmed in future trials with clearly defined outcome measures.

Regarding safety, 11 of 27 studies reported adverse events such as mild bleeding and pain at the site of needling, fatigue, dizziness, and short-term nausea; however, it appears that these occur less frequently when compared with the medication groups. Additionally, 2 trials reported that when acupuncture was added, it reduced adverse events associated with CC^[22]^ or metformin.^[20]^ Future clinical trials should not neglect to report adverse events associated with acupuncture clearly, including frequency and severity.^[55]^

### 4.3 Strengths and limitations of this review

We acknowledge that there are recently published systematic reviews and meta-analyses.^[12–14]^ However, they showed differences in their results and conclusions. The reasons for these discrepancies may arise from the different search strategies, data extraction, and analysis method. In particular, the Cochrane review has been ignored various hormones related with PCOS.^[12]^ We tried to include key reproductive outcomes associated with PCOS as well as important clinical outcomes including ovulation rate, pregnancy to judge the efficacy, and safety of acupuncture in women with PCOS.

Consistent with other systematic reviews on acupuncture, a big limitation of this report lies in the clinical and methodological diversity of the included studies. PCOS itself is also heterogeneous by nature in terms of clinical and biochemical features. The PCOS phenotypic variability among participating women may have created a variety of clinical manifestations.^[5,56]^ The distribution of age, ethnicity, and BMI can contribute to different manifestations of PCOS.^[56,57]^ Also, some studies adopted CHM as a control group which may not have been standardized. All these clinical as well as methodological diversities and complexities of PCOS may have yielded considerable heterogeneity in our meta-analyses, making generalizability more complicated. On the other hand, only 3 trials reported a formal sample size calculation, and it is of note that most of the included trials are not entirely free from type II error due to small sample sizes.

### 4.4 Implications for further studies

To confirm the ovarian activity, assessments should be conducted more rigorously in future trials. In this review, only 2 studies^[10,44]^ used elevated serum progesterone level >3 ng/mL as indicative of ovulation. Anti-Müllerian hormone (AMH) is positively correlated with the ovarian follicle pool, is elevated in women with PCOS, and has been suggested as a diagnostic tool.^[58,59]^ AMH could be a useful parameter to assess the severity of PCOS^[60]^ and the impact of acupuncture in patients with PCOS.^[58]^ The collection of pre- and posttreatment blood samples at nonstandardized times in the menstrual cycle could be a confounding factor.^[5]^ Moreover, outcome assessment in the majority of trials occurred immediately following the intervention period, and thus we are left with no information on how long acupuncture's effect may persist. More studies with long-term follow-up are needed to examine the effectiveness of acupuncture for improving live births and to assess the sustainability of effect.

Most of the included studies either inadequately reported or did not clearly report methods related to important biases such as randomization/allocation concealment and blinding methods. Future trials should improve their reporting quality by following the Consolidated Standards of Reporting Trials (CONSORT) statement^[61]^ and the Standards for Reporting Interventions in Clinical Trials of Acupuncture (STRICTA).^[62]^

There is an ongoing, large, multicenter RCT from mainland China, Hong Kong, Sweden, and the United States to test the effect of acupuncture with or without CC on live births in women with PCOS.^[9]^ The results of this RCT will add more solid scientific evidence on the effectiveness and safety of acupuncture for patients with PCOS.

## 5 Conclusion

This systematic review and meta-analysis suggests that the evidence base for the use of acupuncture for improving ovulation and menstruation rates and other hormonal changes in women with PCOS is weak. Given the poor reporting and methodological flaws of existing studies, large-scale, long-term RCTs with rigorous methodological input are needed to clarify the role of acupuncture in this population.

## Supplementary Material

Supplemental Digital Content

## Supplementary Material

Supplemental Digital Content
