# Supplementary material for: Acupuncture for polycystic ovarian syndrome: A systematic review and meta-analysis
Source: Medicine (Baltimore). 2017 Jun 8;96(23):e7066. doi: 10.1097/MD.0000000000007066 (PMC5466220; doi:10.1097/MD.0000000000007066)
Supplement: Supplemental Digital Content [file medi-96-e7066-s001.doc]

**Appendix S1.** Search strategy

Appendix S1 Search strategy

We searched electronic databases for relevant studies published through February 2016, comprising four international, three Chinese, six Korean and two Japanese databases. Ovid-Medline (1946 to February 2016); Ovid-EMBASE (1974 to February 2016); Cochrane Central Register of Controlled Trials (CENTRAL); the Allied and Complementary Medicine Database (AMED, 1985 to February 2016); China National Knowledge Infrastructure (CNKI); Wanfang DATA; Chongqing VIP; KoreaMed; Oriental Medicine Advanced Searching Integrated System (OASIS); Korean Medical Database (KMBASE); Korean Studies Information Service System (KISS); Society Database of Korea Institute of Science and Technology Information (KISTI); National Digital Science Library (NDSL); Japan Science and Technology Information Aggregator; Electronic (J-STAGE); and Igaku Chuo Zasshi (ICHUSHI). Combinations of MeSH terms and keywords were used, including “polycystic ovary”, “polycystic ovary syndrome”, “amenorrhea”, “acupuncture”, and “electroacupuncture.” The detailed search strategies are provided below. References of relevant publications (e.g., gynecology textbooks, complementary and alternative medicine textbooks, grey literature, clinical guidelines of infertility, or other review articles) were also hand-searched. No language restrictions were imposed.

|  | **Ovid MEDLINE(R) 1946 to January Week 4 2016** | Date : February 4. 2016 | | |
| --- | --- | --- | --- | --- |
|  | Searches | Results | | |
| **1** | exp Polycystic Ovary Syndrome/ | 10511 | | |
| **2** | polycystic ovary syndrome.tw. | 7102 | | |
| **3** | polycystic ovar$.tw. | 10171 | | |
| **4** | stein-leventhal syndrome.tw. | 544 | | |
| **5** | PCOS.tw. | 5906 | | |
| **6** | PCOD.tw. | 257 | | |
| **7** | hirsut$.tw. | 6168 | | |
| **8** | exp Amenorrhea/ | 9085 | | |
| **9** | exp Oligomenorrhea/ | 643 | | |
| **10** | exp Hirsutism/ | 3384 | | |
| **11** | oligomenorrh$.tw. | 1151 | | |
| **12** | amenorrh$.tw. | 11639 | | |
| **13** | polycystic ovar$ disease.tw. | 747 | | |
| **14** | or/1-13 | 32443 | | |
| **15** | acupuncture.tw. | 13725 | | |
| **16** | exp Acupuncture/ | 1243 | | |
| **17** | exp acupuncture therapy/ | 17148 | | |
| **18** | exp acupuncture, ear/ | 271 | | |
| **19** | exp electroacupuncture/ | 2603 | | |
| **20** | exp meridians/ | 4454 | | |
| **21** | exp acupuncture points/ | 4128 | | |
| **22** | exp moxibustion/ | 1181 | | |
| **23** | electroacupuncture.tw. | 2436 | | |
| **24** | meridian$.tw. | 3677 | | |
| **25** | needling.tw. | 1471 | | |
| **26** | moxi$.tw. | 4743 | | |
| **27** | acup$ point$.tw. | 1150 | | |
| **28** | (shiatsu or tui na).tw. | 71 | | |
| **29** | shu.tw. | 493 | | |
| **30** | acupressure.tw. | 509 | | |
| **31** | (trigger adj3 point$).tw. | 1180 | | |
| **32** | or/15-31 | | | 27907 |
| **33** | 14 and 32 | | | 86 |
| **34** | randomized controlled trial.pt. | | | 383316 |
| **35** | controlled clinical trial.pt. | | 88500 | |
| **36** | randomized.ab. | | 281890 | |
| **37** | placebo.tw. | | 152304 | |
| **38** | clinical trials as topic.sh. | | 170701 | |
| **39** | randomly.ab. | | 199954 | |
| **40** | trial.ti. | | 121398 | |
| **41** | (crossover or cross-over or cross over).tw. | | 56067 | |
| **42** | or/34-41 | | 894078 | |
| **43** | exp animals/ not humans.sh. | | 3982927 | |
| **44** | 42 not 43 | | 818458 | |
| **45** | 33 and 44 | | 35 | |

|  | **Ovid EMBASE 1974 to 2016 January Week 4** | Date : Feb. 4. 2016 | |
| --- | --- | --- | --- |
|  | Searches | Results | |
| **1** | exp ovary polycystic disease/ | | 18048 |
| **2** | polycystic ovary syndrome.tw. | | 10118 |
| **3** | polycystic ovar$.tw. | | 14548 |
| **4** | stein-leventhal syndrome.tw. | | 577 |
| **5** | PCOS.tw. | | 9312 |
| **6** | PCOD.tw | | 325 |
| **7** | hirsut$.tw. | | 8379 |
| **8** | exp amenorrhea/ | | 17484 |
| **9** | exp Oligomenorrhea/ | | 2428 |
| **10** | exp "amenorrhea and oligomenorrhea"/ | | 25130 |
| **11** | exp Hirsutism/ | | 9505 |
| **12** | oligomenorrh$.tw. | | 1624 |
| **13** | amenorrh$.tw. | | 15107 |
| **14** | polycystic ovar$ disease.tw. | | 911 |
| **15** | OR/1-14 | | 56871 |
| **16** | exp Acupuncture/ | | 22003 |
| **17** | acupuncture.tw. | | 33585 |
| **18** | exp electroacupuncture/ | | 4203 |
| **19** | electroacupuncture.tw. | | 1690 |
| **20** | exp moxibustion/ | | 3372 |
| **21** | meridian$.tw. | | 4772 |
| **22** | needling.tw. | | 2207 |
| **23** | moxi$.tw. | | 7068 |
| **24** | acup$ point$.tw. | | 2044 |
| **25** | (shiatsu or tui na).tw. | | 118 |
| **26** | shu.tw. | | 1321 |
| **27** | acupressure.tw. | | 815 |
| **28** | (trigger adj3 point$).tw. | | 2177 |
| **29** | exp acupuncture needle/ | | 289 |
| **30** | exp acupressure/ | | 1344 |
| **31** | exp Shiatsu/ | | 88 |
| **32** | exp Tui Na/ | | 17 |
| **33** | OR/16-32 | | 47457 |
| **34** | Clinical trial/ | | 843067 |
| **35** | Randomized controlled trial/ | | 360546 |
| **36** | Randomization/ | | 64567 |
| **37** | Single blind procedure/ | | 1938 |
| **38** | Double blind procedure/ | | 119901 |
| **39** | Crossover procedure/ | | 41319 |
| **40** | Placebo/ | | 264346 |
| **41** | Randomi?ed controlled trial$.tw. | | 108708 |
| **42** | Rct.tw. | | 15816 |
| **43** | Random allocation.tw. | | 1402 |
| **44** | Randomly allocated.tw. | | 21700 |
| **45** | Allocated randomly.tw. | | 2002 |
| **46** | (allocated adj2 random).tw. | | 801 |
| **47** | Single blind$.tw. | | 15432 |
| **48** | Double blind$.tw. | | 153317 |
| **49** | ((treble or triple) adj blind$).tw. | | 443 |
| **50** | Prospective study/ | | 273483 |
| **51** | OR/34-50 | | 1414203 |
| **52** | Case study/ | | 30140 |
| **53** | Case report.tw. | | 282500 |
| **54** | Abstract report/ or letter/ | | 931848 |
| **55** | 52 or 53 or 54 | | 1238398 |
| **56** | 51 NOT 55 | | 1375337 |
| **57** | 15 AND 33 AND 56 | | 99 |

|  | **Ovid AMED (Allied and Complementary Medicine) 1985 to January 2016** | **Date : Feb. 4. 2016** |
| --- | --- | --- |
|  | Searches | Results |
| **1** | exp Polycystic Ovary Syndrome/ | 10 |
| **2** | polycystic ovary syndrome.tw. | 42 |
| **3** | polycystic ovar$.tw. | 56 |
| **4** | stein-leventhal syndrome.tw. | 1 |
| **5** | PCOS.tw. | 28 |
| **6** | PCOD.tw. | 14 |
| **7** | hirsut$.tw. | 64 |
| **8** | exp Amenorrhea/ | 34 |
| **9** | oligomenorrh$.tw. | 9 |
| **10** | amenorrh$.tw. | 88 |
| **11** | polycystic ovar$ disease.tw. | 7 |
| **12** | or/1-11 | 208 |
| **13** | acupuncture.tw. | 9168 |
| **14** | exp Meridians/ | 439 |
| **15** | exp Electroacupuncture/ | 782 |
| **16** | exp Acupuncture/ | 3248 |
| **17** | exp Acupoints/ | 1572 |
| **18** | exp Acupuncture therapy/ | 7118 |
| **19** | exp Ear acupuncture/ | 396 |
| **20** | exp Moxibustion/ | 497 |
| **21** | electroacupuncture.tw. | 922 |
| **22** | meridian$.tw. | 620 |
| **23** | needling.tw. | 781 |
| **24** | moxi$.tw. | 591 |
| **25** | acup$ point$.tw. | 594 |
| **26** | exp acupressure/ | 278 |
| **27** | exp Shiatsu/ | 270 |
| **28** | (shiatsu or tui na).tw. | 326 |
| **29** | shu.tw. | 84 |
| **30** | acupressure.tw. | 356 |
| **31** | (trigger adj3 point$).tw. | 409 |
| **32** | or/13-31 | 10766 |
| **33** | 12 and 32 | 24 |

|  | **Cochrane Central Register of Controlled Trials : Issue 1 of 12, January 2016** | Date : Feb. 4. 2016 | |
| --- | --- | --- | --- |
|  | Searches | Results | |
| **1** | MeSH descriptor: [Polycystic Ovary Syndrome] explode all trees | 952 | |
| **2** | polycystic ovary syndrome | 1597 | |
| **3** | polycystic ovar* | 1831 | |
| **4** | stein-leventhal syndrome | 18 | |
| **5** | PCOS | 1277 | |
| **6** | PCOD | 41 | |
| **7** | Hirsut* | 553 | |
| **8** | MeSH descriptor: [Amenorrhea] explode all trees | 275 | |
| **9** | MeSH descriptor: [Oligomenorrhea] explode all trees | 34 | |
| **10** | MeSH descriptor: [Hirsutism] explode all trees | 184 | |
| **11** | Oligomenorrh* | 182 | |
| **12** | Amenorrh* | 1333 | |
| **13** | polycystic ovar* disease | 678 | |
| **14** | #1 or #2 or #3 or #4 or #5 or #6 or #7 or #8 or #9 or #10 or #11 or #12 or #13 | 3562 | |
| **15** | MeSH descriptor: [Acupuncture] explode all trees | 159 | |
| **16** | acupuncture | 9825 | |
| **17** | MeSH descriptor: [Acupuncture Therapy] explode all trees | 3772 | |
| **18** | MeSH descriptor: [Acupuncture, Ear] explode all trees | 157 | |
| **19** | MeSH descriptor: [Electroacupuncture] explode all trees | 596 | |
| **20** | MeSH descriptor: [Meridians] explode all trees | 1426 | |
| **21** | MeSH descriptor: [Acupuncture Points] explode all trees | 1395 | |
| **22** | MeSH descriptor: [Moxibustion] explode all trees | 311 | |
| **23** | electroacupuncture | 1281 | |
| **24** | Meridian* | 766 | |
| **25** | needling | 1065 | |
| **26** | Moxi* | 4198 | |
| **27** | Acup* point* | 3720 | |
| **28** | (shiatsu or tui na) | 61 | |
| **29** | shu | 853 | |
| **30** | acupressure | 691 | |
| **31** | (trigger adj3 point*) | 166 | |
| **32** | MeSH descriptor: [Acupressure] explode all trees | | 266 |
| **33** | #15 or #16 or #17 or #18 or #19 or #20 or #21 or #22 or #23 or #24 or #25 or #26 or #27 or #28 or #29 or #30 or #31 or #32 | | 12723 |
| **34** | #14 AND #33 in Trials | | 40 |

|  | **CNKI** | Date : : Feb. 4. 2016 |
| --- | --- | --- |
|  | Searches | Results |
| **#1** | (SU='针刺' or SU='电针' or SU='耳针'or SU='针灸'or SU='针'or SU='温针灸'or SU='腹针'or SU='穴位埋线'or SU='芒针') and (SU='多囊卵巢综合征'or SU='PCOS'or SU='多囊卵巢'or SU='polycystic ovary') | 468 |

|  | **CQVIP** | Date : : Feb. 4. 2016 |
| --- | --- | --- |
|  | Searches | Results |
| **#1** | 题名或关键词=多囊卵巢综合征 或者 题名或关键词=PCOS 或者 题名或关键词=多囊卵巢 或者 题名或关键词=polycystic ovary 并且 题名或关键词=针刺 或者 题名或关键词=针灸 或者 题名或关键词=温针灸 或者 题名或关键词=电针 或者 题名或关键词=腹针 或者 题名或关键词=耳针 或者 题名或关键词=芒针 或者 题名或关键词=针 或者 题名或关键词=穴位埋线 | 188 |

|  | **Wanfang** | Date : : Feb. 4. 2016 |
| --- | --- | --- |
|  | Searches | Results |
| **#1** | (题名或关键词:(多囊卵巢综合征) + 题名或关键词:(PCOS)+题名或关键词:(多囊卵巢)+题名或关键词:(polycystic ovary)) * 题名或关键词:(针刺+针灸+温针灸+电针+腹针+耳针+芒针+针+穴位埋线) | 255 |

|  | **J-Stage** | Date : : Feb. 4. 2016 |
| --- | --- | --- |
|  | Searches | Results |
| **#1** | acupuncture AND Full Text: PCOS | 4 |

|  | **ICHUSHI** | Date : Feb. 4. 2016 |
| --- | --- | --- |
|  | Searches | Results |
| **#1** | (多嚢胞性卵巣症候群/TH or PCOS/AL) | 2,541 |
| **#2** | (多嚢胞性卵巣症候群/TH or 多嚢胞性卵巣症候群/AL) | 2,585 |
| **#3** | ((鍼療法/TH or acupuncture/AL) or (鍼灸医学/TH or acupuncture/AL)) | 15,341 |
| **#4** | (鍼療法/TH or 鍼/AL) | 30,277 |
| **#5** | #3 or #4 | 30,403 |
| **#6** | (鍼灸医学/TH or 鍼灸医学/AL) | 2,697 |
| **#7** | #5 or #6 | 30,403 |
| **#8** | #1 or #2 | 2,630 |
| **#9** | #5 and #8 | 6 |

|  | **OASIS (Korea)** | Date : : Feb. 4. 2016 |
| --- | --- | --- |
|  | Searches | Results |
| **#1** | 다낭성 | 30 |

|  | **KISS (Korea)** | Date : : Feb. 4. 2016 |
| --- | --- | --- |
|  | Searches | Results |
| **#1** | 다낭성 AND 침 | 2 |
| **#2** | PCOS AND 침 | 0 |
| **#3** | PCOS AND acupuncture | 0 |
| **#4** | 다낭성 AND acupuncture | 2 |

|  | **NDSL (Korea)** | Date : : Feb. 4. 2016 |
| --- | --- | --- |
|  | Searches | Results |
| **#1** | BI:((침 or acupuncture) AND (PCOS or 다낭성)) | 7 |

|  | **Koreamed (Korea)** | Date : : Feb. 4. 2016 |
| --- | --- | --- |
|  | Searches | Results |
| **#1** | PCOS [ALL] AND acupuncture [ALL] | 0 |

|  | **KISTI Society (Korea)** | Date : : Feb. 4. 2016 |
| --- | --- | --- |
|  | Searches | Results |
| **#1** | ((BI : PCOS) OR (BI : 다낭성)) AND ((BI : 침) OR (BI : ACUPUNCTURE))) | 7 |

|  | **KMBASE (Korea)** | Date : : Feb. 4. 2016 |
| --- | --- | --- |
|  | Searches | Results |
| **#1** | PCOS AND 침 | 1 |
| **#2** | 다낭성 AND 침 | 10 |

**Appendix S2.** Study selection

Appendix S2. Study selection

Types of studies

We sought RCTs that evaluated the effects of acupuncture in the treatment of PCOS. Non-randomized trials, quasi-experimental studies, and observational studies were excluded. Animal studies, qualitative studies, letters, news articles, editorials, and commentaries were also excluded.

Types of participants

The inclusion criterion for participants was diagnosis of PCOS. The diagnostic criteria adopted in our review were based on the European Society of Human Reproduction and Embryology (ESHRE) and the American Society of Reproductive Medicine (ASRM) consensus in Rotterdam 2003 (ESHRE/ASRM 2004) (1). In summary, a woman with two of the three following criteria was considered as having PCOS:

oligo or anovulation;

hyperandrogenemia; or

polycystic ovaries.

The exclusion criteria were presence of other etiologies of hyperandrogenism such as hypothyroidism, androgen secreting tumor, hyperprolactinemia, Cushing syndrome, and congenital adrenal hyperplasia.

Types of interventions

We included only trials in which acupuncture needles were inserted (and manipulated) into traditional acupoints or tender points (i.e., ashi points) with or without electrical stimulation. Acupuncture-related techniques that did not involve skin penetration, such as acupressure or moxibustion, were not considered. Studies investigating the combined effects of acupuncture with other modalities, such as herbal medicine or laser acupuncture, were also excluded.

Types of control groups

We sought studies adopting sham acupuncture, no treatment, or other active treatments, as control groups. Active treatments included weight loss through lifestyle modifications and medications. Studies comparing acupuncture as an adjunct to standard care were also considered, when standard treatments were applied to both acupuncture and control groups in an identical manner.

Types of outcome measures

Primary outcomes were monthly ovulation and menstrual rates. The diagnostic criterion for ovulation was determined by ultrasound or increased progesterone level. The secondary outcomes included LH, LH/FSH ratio, testosterone levels, fasting insulin levels, pregnancy rate, and adverse events.

**Appendix S3.** The authors’ judgment on risk of bias

Appendix S3. The authors’ judgment on risk of bias

Random sequence generation

Among the 27 RCTs, four studies employed inadequate randomization procedures based on an admission order^25, 27, 31, 34^, while 13 studies^10, 21-24, 26, 28, 36, 38, 42-45^ described adequate methods of random sequence generation, including computer-generated randomization and randomization tables. We rated the other ten studies^20, 29, 30, 32, 33, 35, 37, 39-41^ as having an unclear risk of bias because they failed to describe the method of random number generation used.

Allocation concealment

Three trials^22, 44, 45^ using sequentially numbered sealed envelopes were given a low risk of bias based on allocation concealment, while seven^21, 24, 25, 27, 31, 34, 36^ were at high risk of bias because the investigators enrolling participants could possibly foresee assignments and thus introduce selection bias. We rated the other 17 studies^10, 20, 23, 26, 28-30, 32, 33, 35, 37-43^ were at unclear risk of bias because they did not describe any method of allocation concealment.

Blinding of participants and personnel

For participant blinding, eight studies adopting sham acupuncture as control groups were considered to have a low risk of bias.^20, 23, 32, 35, 37, 38, 44, 45^ The rest of the included studies were given a high risk of bias because the participants could not be blinded when acupuncture was compared with active medication or no treatment control.

Blinding of outcome assessment

Four trials^10, 43-45^ were at low risk of bias. Jedel *et al*.(2011) reported that investigators analyzing data were blinded to treatment allocation and were not involved in the randomization procedure. Johansson *et al.* (2013) reported that investigators were blinded until statistical analyses was completed and all the outcomes were measured at an independent laboratory. Lim *et al.* (2014) reported that outcome assessment was done by statisticians who were independent of the study. Pastore *et al*.(2011) reported that the principal investigator, clinical research coordinator, and biostatistician were all blinded to the intervention arm. We rated the other 23 studies as having an unclear risk of bias because insufficient information was provided to determine whether or not investigators were blinded.

Incomplete outcome data

One trial^44^, which did not report the reason for drop-outs and did not perform the analysis based on intention-to-treat (ITT), was given a high risk of bias. Five^10, 22, 24, 43, 45^ described reasons for drop-outs adequately in the published reports and drop-out reasons were similar and balanced across groups, so we considered them as having a low risk of bias. Two trials^38, 42^ reported that no participants dropped out or were lost to follow-up during intervention, so we classified them as having a low risk of bias. Those that did not report missing data or drop-outs, were considered as having an unclear risk of bias.

Selective outcome reporting

Two trials^28, 44^ were at high risk of bias for selective reporting because they presented incomplete hormone data. The other trials were at low risk of bias as they reported all expected outcomes.

Other sources of bias

There was a low risk of other sources of bias based on lack of clear evidence.
