# Supplementary material for: Acupuncture for polycystic ovarian syndrome: A systematic review and meta-analysis
Source: Medicine (Baltimore). 2017 Jun 8;96(23):e7066. doi: 10.1097/MD.0000000000007066 (PMC5466220; doi:10.1097/MD.0000000000007066)
Supplement: Supplemental Digital Content [file medi-96-e7066-s002.docx]

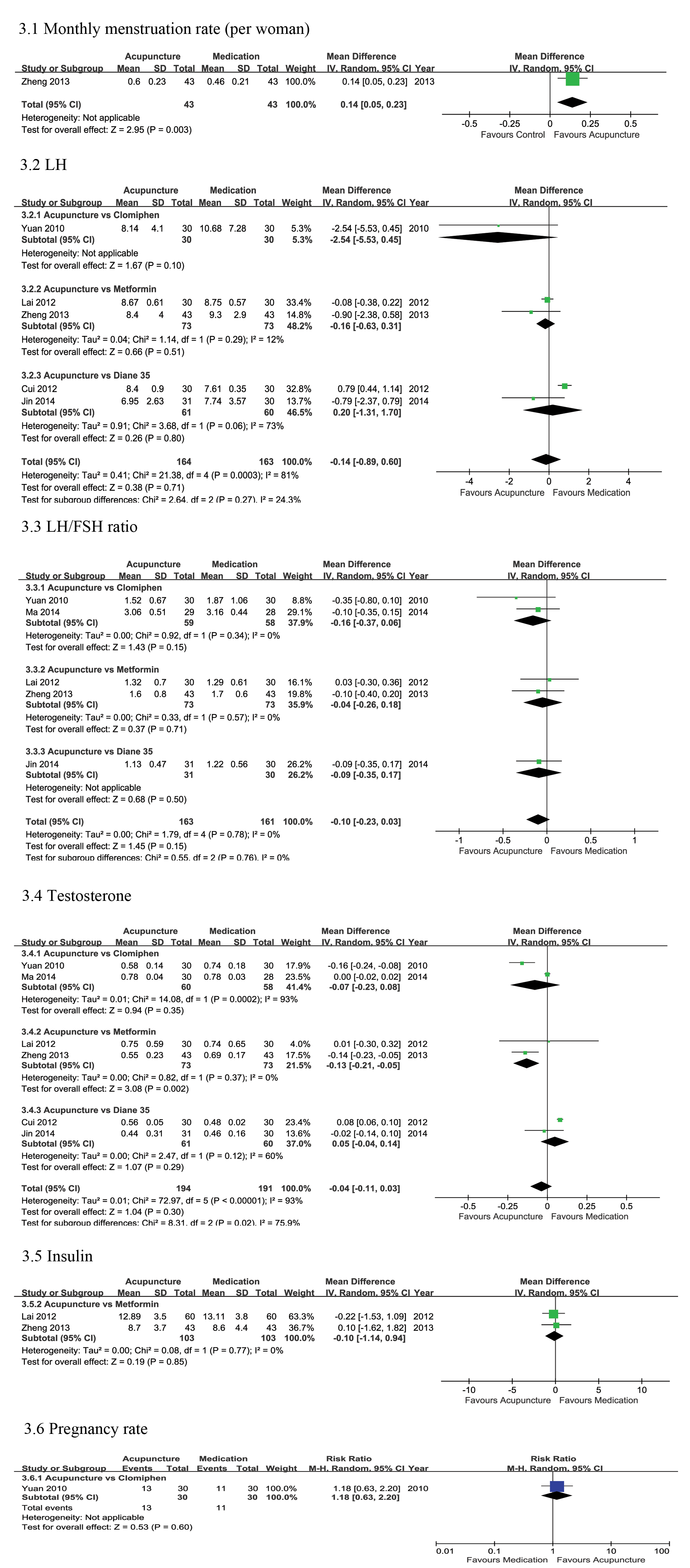


**Figure S1.** Forest plot of outcomes in women with PCOS comparing acupuncture versus medication


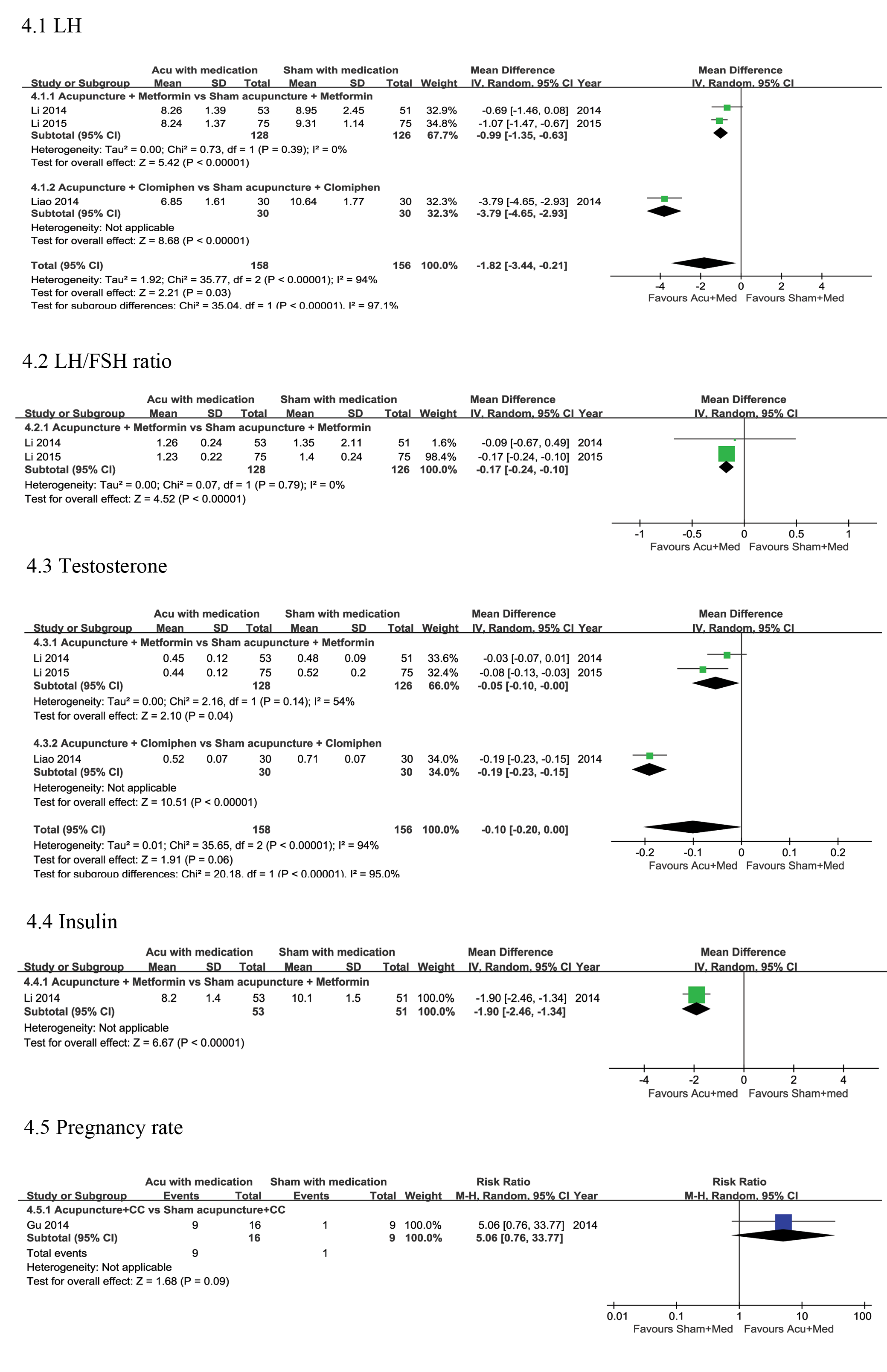


**Figure S2.** Forest plot of outcomes in women with PCOS comparing acupuncture with medication versus sham acupuncture with medication
